# Alström Syndrome: A Challenging Case Study of a Female Saudi Patient With Type 2 Diabetes Mellitus and Complete Vision Loss

**DOI:** 10.7759/cureus.60396

**Published:** 2024-05-15

**Authors:** Abdulrahman S Alamri, Hatim A Mahmoud, Abdulaziz A Abu Alnasr, Alaa K Alahmadi, Yousef H Qari

**Affiliations:** 1 Diabetes and Endocrinology Department, Prince Mohammed Bin Abdulaziz National Guard Hospital, Madinah, SAU; 2 Department of Family Medicine, Prince Mohammed Bin Abdulaziz National Guard Hospital, Madinah, SAU; 3 Neuroscience Department, King Abdullah Medical Complex, Ministry of Health - Kingdom of Saudi Arabia, Jeddah, SAU

**Keywords:** saudi, chronic kidney disease, double diabetes, alström syndrome, alms1

## Abstract

Alström syndrome is a genetic disease that impacts numerous systems in the human body. The symptoms can vary and appear gradually. Childhood obesity, heart disease (cardiomyopathy), abnormalities in vision, and hearing issues are the main symptoms of this disorder in children. Diabetes mellitus, hepatic issues, and renal dysfunction can all occur over time. Genetic alterations in the ALMS1 gene are the cause of Alström syndrome. It has an autosomal recessive inheritance pattern. We address the case of a Saudi woman in her 20s. She had been initially referred for type 2 diabetes, intellectual disability since early childhood, metabolic acidosis, and micrognathia; however, she also exhibited blindness, chronic kidney disease (CKD), and hearing loss, all of which are indicative of Alström syndrome. DNA testing showed that she has a homozygous pathogenic variant in the ALMS gene. Autosomal recessive Alström syndrome has been confirmed as a genetic diagnosis. No other clinically significant variations were found that are associated with the mentioned phenotype. By reporting this mutation, we hope to learn more about the genotypic range of the disease, particularly in the Saudi population. As each member of the family underwent genetic testing, we established a stringent follow-up schedule for our patient and her family.

## Introduction

Alström syndrome (AS; OMIM 203800) is an extremely rare autosomal recessive disease [1,2]. The disease was initially documented in 1959 in a Swedish family [3]. An estimated one to nine cases of ALMS occur for every million people [4]. Childhood obesity, sensorineural hearing loss, progressive cone-rod dystrophy resulting in blindness, adult low height with early onset rapid childhood linear growth are the hallmarks of AS [5]. Early onset diabetes mellitus (usually developing in the second or third decade), hyperinsulinemia, hypothyroidism, infertility (hypergonadotropic hypogonadism), and hypertriglyceridemia are examples of endocrinologic complications [6-7]. In addition, it is typical for systemic fibrosis to occur in AS [5]. Renal failure is the leading cause of death in older subgroup of the affected patients, whereas in young patients, dilated cardiomyopathy-related cardiac involvement is the primary cause of mortality [8].

The ALMS1 gene, which is found on chromosome 2p13, encodes a 4169-amino acid protein that includes a large 47-amino acid tandem-repeat domain [9]. The ALMS1 gene contains 23 exons and produces a 4,169 amino acid, 461 kDa protein. The ALMS1 protein is widely expressed and found at the basal bodies and centrosomes of ciliated cells, indicating that it may play a role in endosomal recycling, cellular migration, intraciliary transport, cell division regulation, and cytoskeleton maintenance [10]. Nevertheless, the precise function of the ALMS1 protein is still unknown [10]. It has been demonstrated that the ALMS1 protein localizes subcellularly and is expressed widely [9]. It has been suggested that ALMS1 plays a role in basal body or centrosome function [10]. Furthermore, it is established that mutations in the ALMS1 gene, which produces the ALMS1 protein, are the main cause of AS [10]. Although AS patients can exhibit their first clinical symptoms as early as infancy, there can be a significant variation in the actual age of onset and severity of symptoms, even between relatives with identical ALMS1 variants [11]. The rapid advancement of molecular analysis technology has made an early diagnosis possible. Here, we describe a patient who developed poor vision, sensorineural deafness, CKD, diabetes mellitus, intellectual disability, metabolic acidosis, and micrognathia as a result of mutations in the ALMS1 gene. Whole-exome sequencing in conjunction with a urine analysis test has aided in establishing the diagnosis [12].

## Case presentation

A 21-year-old Saudi woman, born to consanguineous parents, presented to the emergency department at Prince Muhmmed Bin Abdulaziz National Guard Hospital in Al Madinah, complaining of left lumbar pain that started suddenly 4 days prior to presentation and progressed over time which was relieved with simple analgesics. She reported subjective fever associated with poor oral intake, although when checked in the primary health care center, no fever was documented. Moreover, she experienced, non-bloody, non-bilious, watery diarrhea four times a day. The patient has reported dysuria, with decreased urine output and hematuria. No previous history of nephrotoxic medications or similar presentations was noted before. Other systemic reviews were unremarkable.

Her prior medical history was significant for poor vision since the age of 3 months, which began with the development of night blindness and decremental loss of hearing. As a result, the patient performed poorly at school. She was not known to have diabetes or kidney disease upon admission. The patient had no prior history of symptoms suggestive of diabetes. Family history is positive for a younger sister who is also blind and has hearing loss with intellectual disabilities, further details were not known. Upon admission, the patient’s height was 143 cm, weight 43.5 kg, and her body mass index (BMI) was 21.27 kg/m2. She was diagnosed with acute kidney injury (AKI). Results were notable for a serum creatinine of 615 umol/L (normal range 50-98 umol/L), urine albumin to creatinine ratio (ACR) was 50 mg/g (normal <30 mg/g) measured based on isolated urine sample, (glomerular filtration rate 8 mL/min/1.732) based on the MDRD GFR Equation, sodium was 126 (normal range 135 -145 mEq/L), calcium was 1.97 (normal range 8.6-10.2 mg/dL), and blood urea nitrogen of 29.4 mg/dL (normal range 6.0-21 mg/dL), all of which were confirmed on repeat. The laboratory findings are demonstrated in Table 1.

**Table 1:** Laboratory investigations upon admission

Laboratory Parameters	Upon Admission	Normal Range
Sodium	126	135 and 145 mEq/L
Calcium	1.97	8.6-10.2 mg/dL
Creatinine	615	50-98 umol/L
Urine albumin to creatinine ratio	50	<30 mg/g
Blood urea nitrogen	29.4	6.0–21 mg/dL

**Figure 1:**
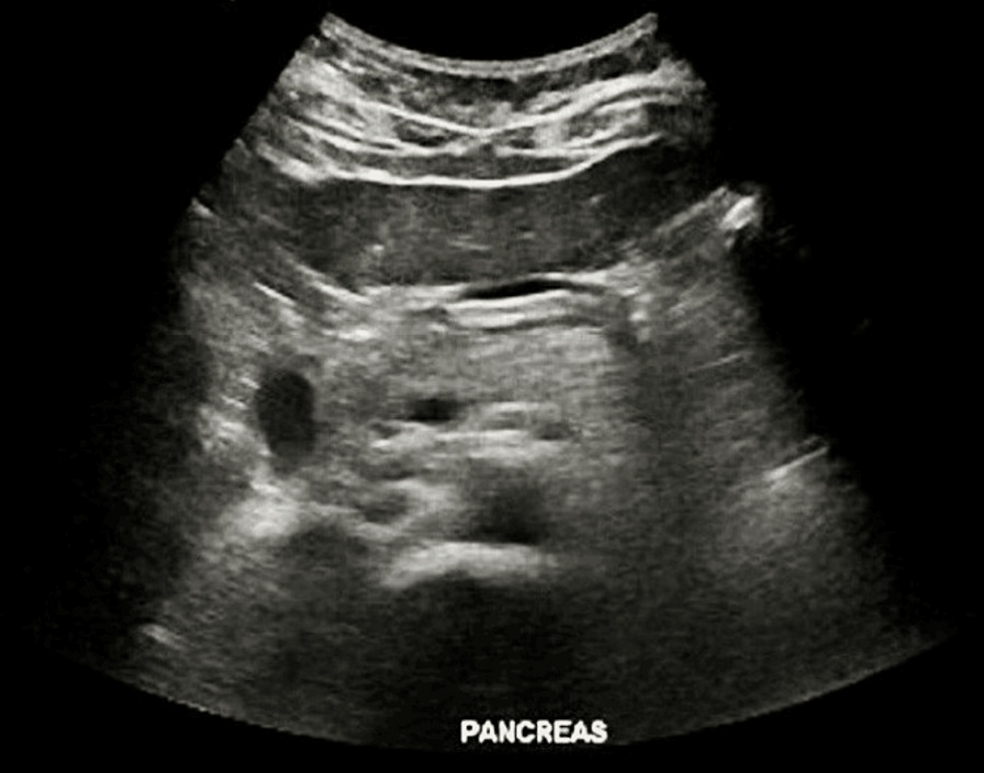
Abdominal ultrasound showing unremarkable gallbladder, no evidence of cholecystitis. Mild diffuse hepatic steatosis

**Figure 2:**
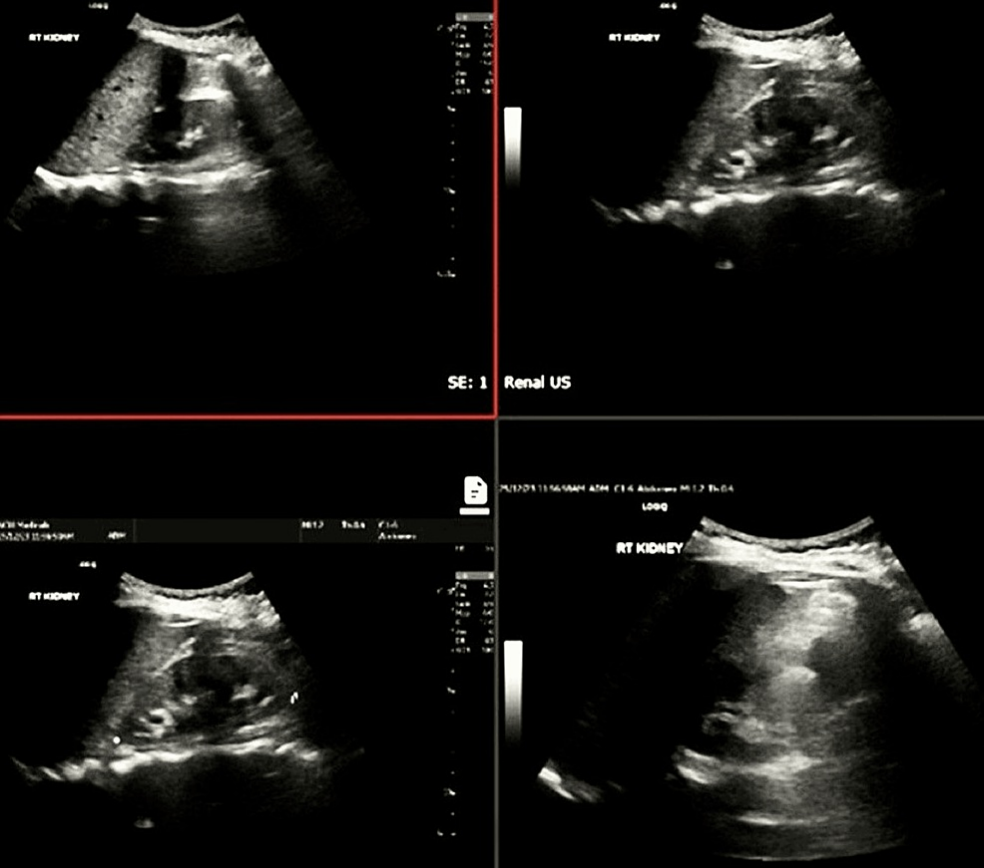
Renal ultrasound showing mild left renal hydronephrosis. Otherwise, unremarkable scan.

Renal ultrasound revealed mild left renal hydronephrosis. Additionally, she suffered from a urinary tract infection (UTI), as evidenced by bacteria and blood in her urine, along with elevated inflammatory markers. Abdominal ultrasound showed mild diffuse hepatic steatosis. Images depicting the previously mentioned studies are shown in Figures 1-2.

The patient completed a course of piperacillin/tazobactam (Tazocin) for a total of 10 days based on the urinalysis result. Eventually, urine and blood cultures showed no growth. The patient was started on Insulin Glargine 8 units and Aspart 3 units based on a type 2 diabetes diagnosis that was made based on the requested laboratory tests upon admission. Her blood sugar was uncontrolled with random fingerstick blood glucose while on insulin therapy being over 600 mg/dL (normal <200 mg/dL) and hemoglobin A1c (HbA1c) being 13.8 (normal 4.4 -6.4%). The C peptide level was 6571 pmol/L (normal 227-971 pmol/L), and the antibodies to glutamic acid decarboxylase (anti-GAD) level was <10 IU/mL (normal <10 IU/mL) all of which excluded autoimmune causes of diabetes. The patient's blood venous gas values were normal and there were no ketones in the urine which excluded the possible diagnosis of diabetic ketoacidosis while her blood sugar readings were high. On examination, the patient was conscious, alert, and oriented. She was not in any respiratory distress and was vitally stable. During a local abdominal examination, she had left lumber tenderness with guarding all over the abdomen. Other systems examination was unremarkable including signs of insulin resistance. She suffered from sensorineural deafness, struggled academically and her eyes showed bilateral horizontal nystagmus. Her echocardiogram reported that the left ventricle was normal in size, with normal left ventricular systolic function (Ejection fraction 55-60%) and no regional wall motion abnormalities. Normal diastolic function was also observed. The right ventricle was normal in size and function, with no hemodynamically significant valvular lesions. During admission, the ophthalmology team assessed the patient, who confirmed the presence of complete vision loss. The endocrine department was consulted because of the abnormal labs of persistent hyperglycemia and elevated parathyroid hormone. Their assessment, initially, raised the suspicion of Wolfram syndrome based on the patient clinical picture and new-onset diabetes. Thus, they asked for a genetic test to confirm the diagnosis. Later, after the patient was discharged and during the follow-up, the results came back revealing Alström Syndrome as a diagnosis.

Treatment

There is no known treatment to stop Alström syndrome's organ involvement from getting worse. Coordinated multidisciplinary care is necessary for Alström syndrome patients in order to develop treatment plans and management strategies. Diabetes is managed by a healthy, low-calorie diet with restricted carbohydrate intake, regular aerobic exercise, and a blood sugar level-adjusted insulin dose. AKI is treated with sodium bicarbonate 650 mg, alfacalcidol 0.5 mcg, and a holistic approach with the nephrology team. For hearing impairment, a myringotomy and/or hearing aids may be necessary.

## Discussion

Alström syndrome (OMIM 203800) is an autosomal recessive disorder characterized by cone-rod dystrophy, obesity, progressive bilateral sensorineural hearing loss, and insulin resistance/type 2 diabetes mellitus (T2DM). Also, the disease presents with acute restrictive cardiomyopathy that is of adult-onset, infantile-onset and/or adolescent cardiomyopathy, chronic progressive kidney disease, nonalcoholic fatty liver disease (NAFLD), all of which are hallmarks of AS [2]. Cone-rod dystrophy typically first manifests as nystagmus, photophobia, and progressive visual impairment between the ages of one and 15 months. By the end of the second decade, many people have completely lost their sense of light, but some people can still read large print well into their third decade [13]. Most babies are born with normal weights, but during the first year of life, they develop truncal obesity. Up to 70% of people have sensorineural hearing loss in their first decade, and by the end of their first or second decade, it may have progressed to severe or moderately severe with a range of (40-70 db). [6]. Acanthosis nigricans is commonly associated with insulin resistance, which progresses to type 2 diabetes in most cases by the third decade.

In our patient, we have been able to detect a homozygous mutation of ALMS1. The genetic foundation of AS patients from Saudi Arabia has been documented in a small number of publications. Genetic linkage mapping and Sanger sequencing were used in the first study, which involved four Saudi AS patients. The results showed allelic heterogeneity of ALMS1 mutations, including c.5534 C>G (S908X) and c.5981delCAGA(1992X) in exon 8, c.8275C >T (R2720X) in exon 10, and IVS18-2 A>T in exon 18 [14]. An ALMS1 gene premature termination codon (p.Arg4052Glyfs*2) is produced by a homozygous frameshift deletion in exon 20 (c.12154_12166del), according to whole-exome sequencing of a 10-year-old Saudi girl who presented with diabetic ketoacidosis, hearing loss, and blindness [15]. A pathogenic mutation in the ALMS1 gene (c.8441C>A, p. S2814*) has been discovered in another whole-exome sequencing study conducted on a 5-year-old Saudi girl who was born into a consanguineous marriage and had photophobia, noticeable nystagmus, and retinal abnormalities with short fingers tapering [16]. According to another retrospective study conducted in Saudi Arabia, 10 distinct ALMS1 mutations (E3649*, Q2648*, p.E913Sfs20, p.Ser2102, p.Arg2928*, p.Ser2102*, p.Arg2722*, p.P3911QfsX16, p.Ser908*, IVS18-3A>T) were found in 19 Alström cases with various ophthalmic phenotypes from 13 Saudi families. Their results supported the idea that children's visual phenotypes should raise the possibility of Alström syndrome [17].

In our case, the patient was severely photophobic and experienced progressive loss of visual acuity since the age of 9. Her eyes showed bilateral horizontal nystagmus, and she experienced hearing loss since the age of 11. She has also manifested sensorineural hearing loss. The severity of visual defects can vary, with some cases showing mild visual impairments [16,17]. Approximately 90% of affected individuals become blind by the age of 16 [2]. Additionally, vision loss may be exacerbated by the development of subcapsular cataracts [2]. There have also been reports of exudative retinopathy in AS. Approximately 80% of those impacted will experience bilateral sensorineural hearing loss in the interim [6]. This is typically characterized by the initial loss of high-frequency sounds and happens later in childhood. Hearing deterioration happens gradually and can occasionally be accompanied by conductive hearing loss as a result of glue ear or chronic otitis media [6]. Early alterations in neurosensory abilities have a profound effect on a child's social development as well as the ability to adapt to their surroundings. While delayed cognitive development is not a common characteristic of AS, approximately 45% of children with AS experience delayed developmental milestones. Generalized sleep disturbances and absence of seizures are possible additional neurological manifestations [1]. It is unknown how frequently psychiatric and mood disorders occur in people with AS. About 50% of people with AS experience renal insufficiency. It is uncertain whether hypertension causes or occurs as a result of renal insufficiency, yet it is present in approximately 30% of affected people. Urge incontinence, poor flow, urine retention, and difficulty in starting to void are common signs of urologic dysfunction in both men and women. AS can also result in urologic anatomical anomalies such as dilated ureters, constricted ureteropelvic angles, calyceal deformities, and kidney misalignment [18]. The defining feature of AS is renal disease, which begins early and worsens with age to produce a high prevalence of advanced CKD in children. When making a differential diagnosis for uncommon genetic renal diseases, AS should be taken into account.

In this case, diabetes has developed in her second decade of life, with high blood glucose requiring insulin. Eighty percent of people over 16 have been diagnosed with type 2 diabetes, insulin resistance, and hyperinsulinemia. All of those have been shown in people as young as 1 year old, even before obesity might manifest [6]. Characteristic facial features frequently associated with AS include a round face, deep-set eyes, thick ears, dental anomalies, hyperostosis frontalis interna, and premature frontal balding. Their feet are usually described as being thick and wide, while their fingers and toes are usually short and stubby with neither polydactyly nor syndactyly [5] As mentioned before, we found that our patient had micrognathia. In 1991, hepatic involvement in AS was first reported. Hepatic involvement may occur in about 80% of AS patients, with symptoms ranging from a slight increase in liver transaminases to hepatic steatosis to overt cirrhosis with portal hypertension [19].

## Conclusions

This study presents a Saudi female patient with Alström syndrome confirmed by genetic testing that verified the autosomal recessive inheritance of an extremely rare ALMS1 mutation. The disease's early-life complications are most likely brought on by malfunctioning cilia. Alström syndrome symptoms can impact a wide range of organ systems and vary greatly in presentation and severity. The following conditions can lead to complications that include hepatic, renal, sensory, endocrine, and renal. Alström syndrome does not currently have a specific treatment; instead, current care focuses only on managing the condition's complications. The effects of Alström syndrome are profound for those who are afflicted, and further study should be done in this area. To sum up, we believe that this paper will add to the available literature of patients with Alström syndrome in Saudi Arabia.
